# Risky sexual behaviours among women of reproductive age in a high HIV burdened township in KwaZulu-Natal, South Africa

**DOI:** 10.1186/s12879-020-05302-1

**Published:** 2020-08-01

**Authors:** Mbuzeleni Hlongwa, Karl Peltzer, Khumbulani Hlongwana

**Affiliations:** 1 Discipline of Public Health, School of Nursing and Public Health, University of KwaZulu-Natal, Durban, South Africa; 2 HIV/AIDS/STIs and TB Research Programme, Human Sciences Research Council, Cape Town, South Africa; 3 Department of Research Administration and Development, University of Limpopo, Turfloop, South Africa

**Keywords:** Sexual behaviour, Condom use, HIV, STIs, Women, KwaZulu-Natal, South Africa

## Abstract

**Background:**

Despite several intervention programmes in South Africa, risky sexual behaviours among women of reproductive age remain a public health concern, making them vulnerable to unintended pregnancies and/or sexually transmitted infections (STIs), including human immunodeficiency virus (HIV) infection. The aim of this study was to investigate the predictors of risky sexual behaviours among women of reproductive age in a high HIV-burden township in KwaZulu-Natal, South Africa.

**Methods:**

In a cross-sectional study, 471 women of reproductive age (18–49 years, mean: 25.83) in 10 public health clinics in Umlazi Township, responded to a structured questionnaire. Data were coded, entered into Epi Data Manager and exported to Stata for analysis. A Pearson Chi-square tests and logistic regression models (bivariate and multivariate) were employed to assess the level of the association between the predictor and outcome variables and the *p*-value < 0.05 was considered statistically significant.

**Results:**

More than half (51.80%) of the women were aged 18–24 years and only a handful (18.26%) had a tertiary qualification. The majority were single (88.96%) and the unemployed accounted for 53.50%. This study found that women who had talked about condoms with their partner in the past 12 months were more likely (p = < 0.0001) to have used condoms during their last sexual intercourse. Older women (*p* = 0.035) were more likely to have used a condom at last sex, compared to younger women. However, women who were exposed to physical partner violence (hitting and/or slapping), those who had been diagnosed with HIV and those whose sexual partners were diagnosed with HIV, did not show a significant association with condom use at last sex.

**Conclusion:**

Exposure to physical partner violence and poor partner discussions about condoms are key deterrents to condom usage. Holistic interventions are required in order to address the risky behaviours, and consequently reduce sexually transmitted infections and/or unintended pregnancies.

## Background

Despite several intervention programmes to curb the spread of sexually transmitted infections (STIs), such as HIV, in South Africa, risky sexual behaviours among women of reproductive age remain a public health concern. Risky sexual behaviours expose women to unintended pregnancies and STIs [1–3]. Many negative health outcomes such as unintended pregnancies and STI infections among women, have been linked to risky sexual habits [4]. These include, s having multiple sexual partners, engaging in unprotected sex and having sex under the influence of drugs or alcohol [5, 6]. While about one-fifth of women in their reproductive age (15–49 years) in South Africa are HIV positive [7], an increasing number of adolescents with early sexual debut, multiple sexual partners and inconsistent condom use has also been observed [3, 8]. Poor sexual communication has been flagged as one of the most important challenges facing these younger women [6], compounded by their vulnerability to gender-based violence (GBV) from their male partners [3, 9]. This, in turn, limits their ability to negotiate for safer sex [10]. These risky sexual behaviour patterns continue to rise in South Africa, with reports showing an increasing number of people with multiple sexual partners and inconsistent condom use at last sex [8, 11].

The South African government has developed and implemented many interventional programmes and strategies, such as the National Adolescent Sexual and Reproductive Health and Rights Framework Strategy (2014–2019) and The National HIV, AIDS and STI Strategic Plan for South Africa 2007–2011 [12, 13], aimed at educating and encouraging safer sexual practices. Although these intervention programmes and strategies put emphasis on improving the sexual behaviours of South African women, risky sexual behaviours still persist. Studies conducted in other parts of South Africa with similar settings, on the predictors of risky sexual behaviours among women, produced mixed findings. While increased access to HIV testing services has yielded some positive influence on risky sexual behaviour [14], on the same vein, there has been reports that utilizing HIV testing services has no influence on sexual behaviours of individuals [15].

Notably, KwaZulu-Natal (KZN) province has the highest HIV prevalence in South Africa among people aged 15–49 years (27%) [11], and 31.6% among women aged 20–24 years [8]. Therefore, conducting this study in this region provides an important opportunity to understand the predictors of risky sexual behaviours among women from a clinic-based setting. Sexual behaviour in the context of this study is defined as a form of sexual encounter with a single or multiple sexual partners, and including the use or non-use of preventive measures against sexually transmitted infections and/or pregnancy. On the other hand, risky sexual behaviour includes inconsistent condom use during the last sexual encounter. This study investigated the predictors of condom use at last sex among women of reproductive age in a high HIV-burden township in KZN, South Africa, using a cross-sectional survey. The findings of this study are expected to be useful for informing policy-makers, healthcare professionals and researchers on the predictors of risky sexual behaviours.

## Methods

### Study setting

Umlazi Township, which is located in KZN province, is the second most populated township in South Africa, with an estimated population of more than half a million people [16]. The Township falls under the EThekwini Metro, which has the highest HIV prevalence in South Africa [11]. Umlazi has 10 public clinics serving on average a total of more than 50,000 clients per month, and one public hospital. All 10 public health clinics participated in the study.

### Study design, participants and sampling

An analytic cross-sectional survey was conducted over a period of 5 months (November 2018 to April 2019). The study sample comprised of 471 women aged 18–49 years who had prior sexual exposure, residing at Umlazi Township and utilising health services at any of the 10 participating clinics located at Umlazi Township. The determination of sample size per each site (clinic) was proportional to the size and volume of patients seen at the clinic. The District Health Information System (DHIS) was used to obtain each clinic’s monthly headcount for the past 6 months preceding data collection, in order to determine the average patient volume. Potentially eligible women who utilised services at the time of data collection were approached, introduced to the study and invited to participate. Women below the age of 18 years and those aged 50 years and older, were excluded. A convenience sampling technique was applied to enrol participants, due to time limitation. To ensure non-interruption of healthcare services, participants were only enrolled in the study after the health services had been rendered and just before they departed from the clinic.

### Study instrument and data collection

A structured questionnaire was designed based on the ideas gleaned from the literature review [17, 18], and translated into both English and IsiZulu languages. The questionnaire was pre-tested on 10 participants who were not going to form part of the actual study. The questionnaire design included sections on the demographic and socioeconomic characteristics, the use of contraceptives and information related to sexual behaviour. Two trained and experienced Research Assistants (RAs), competent in both English and IsiZulu languages administered the questionnaire. The RAs explained the questionnaire in detail to participants who needed support. The inclusion criteria involved (a) women of reproductive age (18–49 years) visiting the healthcare facilities for any services during data collection and (b) women residing in Umlazi Township. Women below the age of 18 years and those aged 50 years and above were excluded. Men of all age-groups and women residing outside of Umlazi Township were also excluded. Data was collected during the clinic’s operating hours (07:00–16:00) from Monday to Friday, after participants had received the services for which they visited the clinics for.

### Ethics

Ethical approval to conduct this study was obtained from the Biomedical Research Ethics Committee (BREC) at the University of KwaZulu-Natal (Ref No: BE424/18). The National Health Research Database (NHRD) from the KwaZulu-Natal Provincial Department of Health (Ref No: KZ_2018009_013), and The EThekwini District’s Ethical Review Committee also approved the study. Gatekeeper permissions were obtained from the participating facilities prior to data collection. To ensure confidentiality of respondents, no personal identifiers were captured in the questionnaires.

### Data analysis

Data were coded, entered into Epi Data Manager (version 4.6) [19] and exported to Stata (version 15.0) [20] for analysis. Data cleaning was conducted to eliminate discrepancies in data before the analysis was carried out. Participants’ background information was analysed using descriptive statistics, while the frequency distribution and cross-tabulations of each predictor and outcome variable was carried out for categorical data. Frequency distributions of continuous variables were tested for normality using Shapiro-Wilks test. Pearson Chi-square tests and logistic regression models (bivariate and multivariate) were employed to assess level of significance and the association between the dependent and exposure variables. A stratified, cluster analysis was used for statistical testing. The Pairwise correlation test was used to assess whether correlations between predictor variables existed. The significance level was kept at *p* < 0.05 for all the analyses, and at a confidence interval of 95%.

## Results

### Background characteristics

The study sample consisted of 471 women who attended clinics in Umlazi Township anytime between November 2018 and April 2019. More than half (*n* = 244, 51.80%) of the participants were aged between 18 and 24 years (Mean: 25.83 and SD: ±6.45). Most women were single (*n* = 419; 88.96%) and of Black African descent (*n* = 464; 98.51%). More than half (*n* = 252; 53.50%) of participants were unemployed, while only a handful (*n* = 86; 18.26%) had completed a tertiary level of education (Table 1).

**Table 1:** Sociodemographic characteristics of participants

Background characteristics of respondents	Categories	Frequency (n)	Percent (%) of respondents by characteristic
**Sex**	Female	471	100.00
**Age group**	18–24 years	244	51,91
	25–34 years	172	36,60
	35–49 years	54	11,49
**Total**		^a^**470**	**100,00**
**Level of education**	Primary	10	2,13
	Secondary	373	79,53
	Tertiary	86	18,34
**Total**		^a^**469**	**100,00**
**Employment status**	Unemployed	252	53,96
	Employed	91	19,49
	Studying	124	26,55
**Total**		^a^**467**	**100,00**
**Marital status**	Married/living with partner	42	9,03
	Single	419	90,11
	Separated	4	0,86
**Total**		^a^**465**	**100,00**
**Population group**	Black African	464	99,15
	Coloured/Asian	4	0,85
**Total**		^a^**468**	**100,00**

^a^The total does not add up to 471 as a result of missing data caused by non-reporting from participants.

### Sexual behaviour among study participants

All (100.0%, *n* = 471) study participants had prior sexual encounter within the past 12 months, with 86.8% (*n* = 409) reporting sexual encounter within the last 3 months preceding the survey (Table 2). More than a third of participants (37.2%, *n* = 175) experienced sexual encounter before reaching 18 years of age. There was an almost equal distribution between women who used a condom at the last sexual encounter (49.7%, *n* = 234) versus those who did not use it (50.3%, *n* = 237) in the past 12 months.

**Table 2:** Sexual behaviour of participants (condom use at last sex)

	Condom use at last sex				
	No (***n*** = 237)		Yes (***n*** = 234)		Total (***n*** = 471)
	n	%	n	%	n
**Number of male sexual partners (past 3 months)**					
0	25	54,3%	21	45,7%	46
1	190	50,4%	187	49,6%	377
More than 1	16	50,0%	16	50,0%	32
**Total**	**231**		**224**		**455**
**Partner employed**					
No	40	50,0%	40	50,0%	80
Yes	172	50,9%	166	49,1%	338
**Total**	**212**		**206**		**418**
**Ever Diagnosed with HIV**					
No	172	54,4%	144	45,6%	316
Yes	49	40,2%	73	59,8%	122
**Total**	**221**		**217**		**438**
**HIV status of sexual partner**					
Negative	145	53,1%	128	46,9%	273
Positive	31	43,7%	40	56,3%	71
Do not know	35	47,9%	38	52,1%	73
**Total**	**211**		**206**		**417**
**Sometimes hitting/slapping with partner**					
Agree	31	63,3%	18	36,7%	49
Neutral	7	63,6%	4	36,4%	11
Disagree	166	49,3%	171	50,7%	337
**Total**	**204**		**193**		**397**
**A lot of trust between you and him**					
Agree	138	50,2%	137	49,8%	275
Neutral	30	58,8%	21	41,2%	51
Disagree	32	50,0%	32	50,0%	64
**Total**	**200**		**190**		**390**
**Partner has control over sex**					
Agree	49	58,3%	35	41,7%	84
Neutral	91	51,1%	87	48,9%	178
Disagree	60	44,8%	74	55,2%	134
**Total**	**200**		**196**		**396**
**Partner has control over condom use**					
Agree	38	55,9%	30	44,1%	68
Neutral	86	50,3%	85	49,7%	171
Disagree	78	49,1%	81	50,9%	159
**Total**	**202**		**196**		**398**
**Diagnosed with STI (past 12 months)**					
No	199	49,6%	202	50,4%	401
Yes	29	53,7%	25	46,3%	54
**Total**	**228**		**227**		**455**
**Sex under influence of alcohol (past 3 months)**					
No	211	50,0%	211	50,0%	422
Yes	16	48,5%	17	51,5%	33
**Total**	**227**		**228**		**455**
**Talked about condoms with partner (past 12 months)**					
No	68	75,6%	22	24,4%	90
Yes	153	43,5%	199	56,5%	352
**Total**	**221**		**221**		**442**
**Age at first sex**					
12–17 years	104	59,4%	71	40,6%	175
18–24 years	117	45,0%	143	55,0%	260
**Total**	**221**		**214**		**435**
**Age group**					
18–24 years	132	54,1%	112	45,9%	244
25–34 years	89	51,7%	83	48,3%	172
35–49 years	16	29,6%	38	70,4%	54
**Total**	**237**		**233**		**470**

More than a quarter of participants (25.9%, *n* = 122) had been diagnosed with HIV positive status in the course of their lives and 40.2% (*n* = 49) of them reported to have not used a condom at their last sex encounter. More than one in six of the participants (15.1%, *n* = 71) were aware of their sexual partners’ HIV positive status. Despite this awareness, more than half of these (56.3%, *n* = 40) used a condom at their last sexual encounter. Only 11.5% (*n* = 54) of participants reported to have been diagnosed with an STI in the past 12 months preceding the study. Of these, 53.7% (*n* = 29) did not use a condom at their last sexual encounter. The majority of participants (89.6%, *n* = 422) did not have sex under the influence of alcohol in the past 3 months.

The majority of participants (71.5%, *n* = 337) reported that they were not exposed to violence with a sexual partner (i.e. sometimes hitting or slapping). About half of these (50.7%, *n* = 171) used a condom at their last sexual encounter. More than a third (36.3%, *n* = 171) of participants were neutral on whether or not the sexual partner has control over condom use, with 50.1% (*n* = 86) of these not having used a condom at the last sex encounter. Close to a quarter (21.2%, *n* = 84) of participants reported that the sexual partner has control over their sexual activities. The majority of participants (74.7%, *n* = 352) had talked about condoms with their partners during the preceding 12 months leading to the study, and 56.5% (*n* = 199) of these used a condom at last sex.

### Factors associated with condom use at last sex encounter in univariate and multivariate analysis

Condom use among women in Umlazi Township showed a significant association with four factors in univariate analysis, and these were: ever diagnosed with HIV (OR 1.78, 95% CI: 1.16–2.72), having talked about condoms with partner during the preceding 12 months (OR 4.02, 95% CI: 2.38–6.80), women’s delayed sexual debut, 18–24 years (OR 1.79, 95% CI: 1.21–2.64) and women’s older age category, 35–49 years (OR 2.80, 95% CI: 1.48–5.29). In multivariate analysis, only two variables were significantly associated with condom use at last sex among women in Umlazi Township. Participants who had talked about condoms with their partner during the preceding 12 months to the study were significantly more likely to have used a condom in their last sex encounter. For example, condom use at last sex was 3.74 times (OR 3.74, 95% CI: 2.01–6.98) more likely in women who had talked about condoms with their partner during the preceding 12 months compared to those who had not. Older women (35–49 years) were significantly more likely to use a condom at last sex compared to their younger counterparts (OR 2.70, 95% CI: 1.07–6.81), suggesting that early sex debut is a risk factor to non-condom usage.

Interestingly, women who reported to have ever been diagnosed with HIV (*p* = 0.466), those whose sexual partners had been diagnosed HIV positive (*p* = 0.847), and those who had been diagnosed with an STI in the past 12 months leading to the study (*p* = 0.139) did not influence condom use at last sex encounter. Women who were exposed to partner violence (i.e. sometimes hitting or slapping with a partner) was not associated with condom use at last sex (*p* = 0.968). Detailed univariate and multivariate analyses results are shown in Table 3.

**Table 3:** Factors associated with condom use at last sex in univariate and multivariate analysis

Determinants	Odds ratios (unadjusted)	***P***-value	95% conf. Interval	Odds ratios (adjusted)	***P***-value	95% Conf. Interval
**Number of male sexual partners (past 3 months)**						
0 (ref)						
1	1.17	0.613	0.63 2.17			
More than 1	1.19	0.705	0.48 2.94			
**Partner employed**	1.00	0.9	0.61 1.63			
No (ref)						
Yes	0.97	0.886	0.59 1.57			
**Ever diagnosed with HIV**						
No (ref)						
Yes	1.78	0.006	1.16 2.72	1.33	0.466	0.62 2.85
**HIV status of sexual partner**				1.50	0.131	0.89 2.53
Do not know (ref)						
Negative	0.81	0.433	0.48 1.36	1.10	0.784	0.57 2.12
Positive	1.89	0.606	0.62 2.29	1.10	0.847	0.44 2.74
**Sometimes hitting/slapping with partner**						
Neutral (ref)						
Agree	1.02	0.982	0.26 3.95	1.03	0.968	0.19 5.55
Disagree	1.80	0.354	0.52 6.27	2.05	0.360	0.44 9.56
**Lots of trust in the relationship**						
(ref)						
Agree	1.42	0.258	0.77 2.60			
Disagree	1.43	0.346	0.68 3.00			
**Partner has control over sex**						
Neutral (ref)						
Agree	0.75	0.275	0.44 1.26			
Disagree	1.29	0.267	0.83 2.02			
**Partner has control over condom use**						
Neutral (ref)						
Agree	0.80	0.436	0.45 1.41			
Disagree	1.05	0.822	0.68 1.62			
**Ever diagnosed with STI (past 12 months)**						
No (ref)						
Yes	0.85	0.574	0.48 1.50	0.59	0.139	0.29 1.19
**Sex under influence of alcohol**						
More than 1 (ref)						
Never	0.84	0.626	0.42 1.68			
**Talked about condoms with partner in 12 months**						
No (ref)						
Yes	4.02	0.000	2.38 6.80	3.74	< 0.0001	2.01 6.98
**Age at first sex**						
(Ref: 12–17 years)						
18–24 years	1.79	0.003	1.21 2.64	1.50	0.150	0.86 2.61
**Age group**						
(Ref: 18–24 years)						
25–34 years	1.10	0.636	0.74 1.62	0.85	0.530	0.52 1.40
35–49 years	2.80	0.002	1.48 5.29	2.70	0.035	1.07 6.81

*Conf*. Confidence

### Discussion

This study aimed to investigate risky sexual behaviours and associated factors among women of reproductive age in Umlazi Township in KZN province, South Africa. The main findings of this study indicate that women who talked about condoms with partner during the preceding 12 months were significantly more likely to use condoms when having sex. Older women (35–49 years) were significantly more likely to use a condom at last sex compared to their younger counterparts, suggesting that early sex debut is a risk factor to non-condom usage. Having been diagnosed with HIV or having a sexual partner with a known HIV positive status, did not show any significant association with condom use at last sex among women in Umlazi Township. The sexual behaviour of women who reported to have more than one sexual partner in the past 3 months was risky, given the inconsistent condom use and exposure to STIs. Women who were exposed to partner violence (i.e. sometimes hit or slapped by a partner) was not significantly associated with condom use at last sex. This finding may be supported by the fact that women who are exposed to partner violence may find it difficult to negotiate condom use.

The findings of this study are consistent with the findings of similar studies conducted in comparable settings [21, 22]. The fact that women who talked about condoms with their partner during the preceding 12 months were more likely to use condoms during their sexual encounter suggests that being in a relationship where women are confident to have discussions related to sexual practices with their partners, is important for improving women’s confidence to negotiate for condom use. Similar findings were shown in a study conducted in Tanzania [21]. Women’s capacity to speak about condoms with their sexual partners provides opportunities for improved sexual behavior and protection against STIs, as well as unintended pregnancies [21], while the opposite may be true for women who are unable to negotiate for condom use.

Despite some similar findings to a study conducted in Tanzania, some aspects are contradictory, in as far as the associations between condom use and multiple sexual partners among women are concerned [21]. However, in Ethiopia, participants who were on antiretroviral therapy (ART) and had multiple sexual partners were more likely to engage in risky sexual behaviour [22], and this pattern was observed in both males and females alike. In this study, we found no evidence to suggest that HIV positive status of women had any significant influence on condom use in multivariate analysis. This supports the importance of strengthening HIV education among women and their sexual partners, given the risks of HIV infection. It has been shown that condom use is effective in preventing the spread of HIV by more than 90% [23].

In a study conducted in South Africa [18], the researchers revealed that factors previously found to be significantly associated with contraceptive use, such as being HIV positive, having been diagnosed with STI in the past 12 months, having concurrent sexual partners and early sexual debut, showed stronger negative associations with contraceptive use among women. Although this study did not precisely focus on contraceptive use, linking these behavioural changing patterns among women is important, given the concerns they are raising. Similar to this study, risky sexual behaviours among participants whose partners were HIV positive was also shown in Ethiopia [22]. There is less chances of condom use at sexual debut among youth [3, 24], suggesting the importance of delaying sexual debut among women until they are able to make the informed and/or guided decisions with full considerations of the exposure to HIV infection [25]. Interventions aimed at encouraging women to delay sexual debut and intentional condom use at first sexual encounter are imperative.

While this study provides an important contribution in the field of sexual and reproductive health, it has notable limitations. Given that the sampling frame for this study was limited to women seeking healthcare services in public health clinics in Umlazi Township, women who do not use public healthcare services or use them less frequently were excluded and/or under-represented in the sample. However, the participants of this study represented all the 10 public health clinics in Umlazi Township. Therefore, the insights gained from the participants will likely be relevant to other public health clinics in similar settings in South Africa. This study sought self-reported sexual health information from participants, thereby making the findings vulnerable to social desirability bias. Furthermore, information deemed to have the potential for leading to judgements may have been withheld by the participants. Some participants may have been unable to recall whether or not a condom was used at last the sexual encounter, leading to incorrect information provided. Information on whether or not HIV positive status was reported among participant’s monogamous partner, was not sought. In addition, the data collection instrument did not capture information on ART use among HIV positive women. Older participants may have been unable to recall the age at which they had sexual debut. This may have contributed to reporting bias. Given the cross-sectional nature of the study design, it is not possible to establish a cause-and-effect relationship between study variables.

The findings of this study raise concerns over women’s exposures to new HIV infections, amid risky sexual behaviours. Therefore, we aim to expand this research project to include a qualitative component towards understanding women’s perceptions and experiences regarding risky sexual behaviours and HIV prevention in Umlazi Township. Conducting longitudinal studies on this topic is important to understand women’s sexual behavioural changes, exposures and patterns over time.

The findings of this study make a case for the importance of implementing and/or strengthening evidence-based educational programmes, aimed at improving women’s sexual behaviours and HIV prevention strategies. We further recommend that such programmes may be integrated into school-health programmes to reach younger women, but also include men.

## Conclusion

Factors associated with risky sexual behaviours among women highlight a great risk of exposure to STIs such as HIV in Umlazi Township, KZN, South Africa. Exposure to physical partner violence and poor partner discussions about condoms are key deterrents to condom usage. Some of the factors previously reported to have significant associations with condom use at last sex among women were not supported by the results of this study. The extent to which women engage in unprotected sexual activities are concerning and highlight an urgent need for a more holistic and adaptable educational approach to improving women’s sexual behaviours, and STIs prevention, as well as including men.

## Data Availability

The datasets used and/or analysed during the current study are available from the corresponding author on reasonable request.

## Abbreviations

AOR: Adjusted odds ratios
CI: Confidence interval
GBV: Gender-based violence
HIV: Human immunodeficiency virus
KZN: KwaZulu-Natal
OR: Odds ratios
SSA: Sub-Saharan Africa
STIs: Sexually transmitted infections
